# Socio-cognitive determinants of safe road-crossing behaviors: an application of the prototype willingness model

**DOI:** 10.5249/jivr.v11i1.1078

**Published:** 2019-01

**Authors:** Mehdi Mirzaei-Alavijeh, Maryam Babakhani, Farzad Jalilian, Masoumeh Vaezi, Fatemeh Jalilian, Shiva Khashij, Behrooz Hamzeh

**Affiliations:** ^*a*^Social Development & Health Promotion Research Center, Kermanshah University of Medical Sciences, Kermanshah, Iran.; ^*b*^Research Center for Environmental Determinants of Health, Kermanshah University of Medical Sciences, Kermanshah, Iran.; ^*c*^Faculty of Medicine, Kateb University, Kabul, Afghanistan.

**Keywords:** Road accidents, Health intervention, Pedestrian, Health Promotion

## Abstract

**Background:**

Pedestrians are one of the most vulnerable groups of road users that potentially are at risk for road traffic injuries and deaths. The present paper reports an application of the Prototype Willingness Model (PWM) to the prediction of road-crossing behaviors among students from Kermanshah University of Medical Sciences (KUMS) in the west of Iran.

**Methods:**

This cross-sectional study was carried out among a sample of 315 medical students who were randomly selected from seven faculties of KUMS in 2017 according to their size, and who filled out a self-administered questionnaire containing a scenario depicting a potentially hazardous road-crossing behavior, followed by items measuring the PWM constructs. Data were analyzed by SPSS version 16 at 95% significant level.

**Results:**

The mean score of safe road-crossing behaviors was 9.57 [95% CI: 9.10, 10.05], ranging from 0 to 16. Attitude, subjective norms, and prototype accounted for 15% and 9% of the variation of willingness and intention, respectively. Willingness was a stronger predictor of the safe road-crossing behaviors (P less than 0.001). The road crossing behavior of female student pedestrian was safer than that of their male counterparts (P less than 0.035).

**Conclusions:**

The results have a number of implications. In particular, PWM-based interventions should focus on willingness in order to encourage safer road-crossing behavior among pedestrians.

## Introduction

Road-crossing injuries represent the most serious threat to life and the second and third leading cause of death in middle/low income and high income countries, respectively.^1^ Due to an increase in motor vehicle ownership, Road Traffic Accidents (RTAs) are increasing, as is traffic volume. The probability of uncontrolled road crossing behavior has also increased. Therefore, reducing road-crossing injuries is one of the most important priorities for governments and their health care systems that needs coordinated efforts.^2^ Overall, pedestrians, cyclists and motorcyclists are the most vulnerable people in road accidents.^3^ The most common cause for damage or injury for pedestrians would be non- or poor compliance with traffic legislation.^4^ Many pedestrians walk on the street and the majority do not adhere to the traffic legislation and cross at intersections illegally.^5^ Evidence shows that the relative risk (RR) of road-crossing injuries in pedestrians who crossed the street with no regard to traffic rules were 8 times more than those who were crossing the street legally and obeying traffic rules.^4^ In fact, accidents occur not merely due to the negligence of drivers, of course, confused pedestrians and unsafe road-crossing decisions are also a key factor.^6^

Nowadays, many road-crossing injuries, especially in the low- and middle-income countries, are increasing due to insufficient regulations, as well as drug and alcohol consumption, an inefficient public health structure, a rapid increase in the number of motor vehicles, careless and untrained drivers and pedestrians, etc.^7^

Statistics indicate that, on average, globally a pedestrian is killed every 2 hours and is injured every 9 minutes in road accidents.^8^ Hence, the World Health Organization (WHO) estimated that pedestrian deaths are 49% which is approximately 1.3 million deaths globally in 2015.^9^ It is expected that the total number of injuries will increase by 65% in 2020 and deaths will rise by more than 80%, if prevention strategies are not implemented.^10^ Thus, governments urgently need to address safe road-crossing decisions and any possible causatively-associated determinants for formulating preventive strategies and intervention that control and reduce this preventable cause of death, injuries and disability.^11^ Various determinants, including environmental determinants (e.g. street type; one or two way), demographic factors (e.g. age), and cognitive determinants (e.g. risk perception) etc. can directly or indirectly affect the pedestrians' road-crossing behaviors.^12,13^ For example, it is estimated that elderly pedestrians over 65 years old account for more than 19% of all pedestrian fatalities, and 8% of all pedestrian injuries, higher than the rates of all the other age groups.^14^

Crossing a road, often a complex task is a critical skill in urban life. One of the key determinants influencing the safety of road crossing decision is the socio-cognitive determinants. In fact, safe road crossing requires both cognitive and physical performance, the cognitive performance is needed for decision making and information processing (e.g. at what moment is it safe to cross the street?) and physical performance which includes gait characteristics (e.g. walking speed).^15^ Furthermore, several studies have indicated that use of cognitive determinants in planning health promotion programs to increase healthy behavior may provide useful results for the promotion of healthy behavior.^16-21^ Using a model to predict a pedestrian's road-crossing decisions, in order to increase the behavioral changes and improve outcomes, is very critical. In this regard, the PWM is one of the most appropriate models to evaluate the risk elements of behavioral decisions such as road crossing behaviors. Based on PWM, people are eager to take a specific behavior (willingness), therefore this willingness is associated with their image, who takes this specific behavior (Prototypes), plus, people's attitudes and also subjective norms predicts intention to take specific behavior and consequently intention predicts actual behavior.^22^

In Iran, with the high incidence rate of RTAs, a combination of the increase in motor vehicles, complex traffic flow, increased traffic volume, lack of pedestrian facilities and also non-compliant drivers, have led to a greater prevalence of road-crossing injuries among pedestrians.^23^

On the other hand, the Iranian government, worried about the high mortality rates from RTIs, decided on a national policy to reduce fatal RTIs in 2004.^24^ This policy might increase the need for determining the road crossing behaviors and the cognitive markers for safe road-crossing decisions.

Therefore; safe road-crossing decisions and any possibly causative associated determinants like socio-cognitive determinants must be examined if appropriate actions are to be developed correctly. The current study was conducted among medical college students in the west part of Iran in 2017, to assess cognitive determinants of safe road-crossing decisions using PWM.

## Methods 

***Theoretical Framework***

The PWM was proposed by Gibbons et al ^23^ according to the PWM, there are two pathways to risk distinctions. A reasoned path mediated by (behavioral) intention/expectation and a social reaction path mediated by (behavioral) willingness. In addition, intention and willingness are predicted by three variables: (a) Attitudes are a person’s positive or negative evaluation of performing the focal behavior; (b) Subjective norms (SN) are a person’s perception of other people’s opinion regarding behavioral performance and (c) Risk image or prototype refers to an image of people who engage in risk behaviors.^9^

***Measure ***

A questionnaire based on the study’s objectives was used for data collection. The validity and reliability of the questionnaire had been tested and established, by obtaining expert opinions, including public health specialists and a behavioral counselor. The questionnaire comprised three parts:

**i:**Socio-demographic data including age (year), level of education MD (Doctor of Medicine), DMD (Doctor of Dental Medicine), Pharmacy and BSc (Bachelor of Science) Students, gender (female, male), parents’ education (primary school, secondary school, diploma, academic), economic status of family - is defined as describing someone’s possessions such as owning a house, furniture, car, etc.- (low, average, good very good), and living in a dormitory (yes, no).

**ii: **To assess a participants’ safe road-crossing behaviors, we used their responses to four questions about the safe road-crossing behaviors including: Do you pay attention to the red light when road-crossing? Do you talk on a cell phone while road-crossing? Do you use the pedestrian bridge when road-crossing? Do you use the crosswalk while road-crossing? Each item was measured on an ordinal 5-point Likert-type scaling (0= never, 4= all time). The reliability coefficient for the safe road-crossing behaviors scale in our study was 0.75, suggesting that the internal consistency was adequate.

**iii: **The items which assessed constructs of the PWM were derived from the questions about safe road-crossing behaviors.^25-27^ There were 20 items which measured the five constructs of 1) attitude, 2) subjective norms, 3) prototype, 4) willingness, and 5) intention. Specifically, five items were used to measure positive attitude towards safe road-crossing behaviors. Five items were designed to measure subjective norms encouraging safe road-crossing behaviors. Five items were to measure positive prototype towards safe road-crossing behaviors. In addition, three items were to measure willingness to adopt safe road-crossing behaviors. Furthermore, two items to measure intention towards safe road-crossing behaviors. In order to facilitate participants’ responses to the items, all items were standardized to a five-point Likert scale, ranging from 1 (very little) to 5 (very much).

***Data collection***

To complete the questionnaires, we contacted the administrative staff in each faculty and found available times when we could distribute the questionnaires among eligible students. Actually, a face-to-face interview lasting 10–12 min was done to complete the questionnaire. The interviewers were trained to ensure that the participants completely understood what they said. Plus, students were helped by the interviewers if they had difficulties understanding the questions. Then we distributed the questionnaires among selected students after we explained the study's aims and obtained the students' consents to participate.

***Statistical analysis***

The Statistical Package for the Social Sciences (SPSS) version 16 was used for the purpose of data entry, manipulation, and analysis. Quantitative variables were expressed as means with SDs, and qualitative/categorical ones as frequencies and percentages. Bivariate correlations were computed to ascertain the magnitude and direction of the associations between the PWM variables and about safe road-crossing behaviors scores. Linear regression analysis (backward method) was performed to explain the variation in the safe road-crossing behaviors on the basis of PWM variables.

***Ethical approval***

The Research Ethics Board of KUMS approved the study protocol (KUMS.REC.1394.445). Further, the participants had been given the participant information statement and had signed the written consent form. Individual personal information was kept confidential.

## Results

The response rate was 95.5%. All 301 students completed the questionnaires (Table 1). The mean age of respondents was 22.3 years [95% CI: 22.1, 22.5], ranged from 19 to 30 years. Most (52.8%) were female and approximately 46.5% (140/301) were BSc students. About 52.2% of the students had average economic status. Nearly 29.9% (90/301) and 29.6% (89/301) of the respondents reported that their father had high school and academic education, respectively. In addition, 37.9% (114/301) of the respondents reported that their mother had primary school. More details of demographic characteristics of the participants are shown in Table 1. As can see in Table 1, sex and safe road-crossing behaviors variables had a significant association. Furthermore, age was not significantly related to safe road-crossing behaviors (r=0.047 & P=0.415).

**Table 1:** Distribution of the demographic characteristics among the participants and association between background variables and safe road-crossing behaviors.

Variables	Number	Percent	Mean (SD)	Sig
**Sex **				
Female	159	52.8	10.05 (4.18)	0.035
Male	142	47.2	9.04 (4.11)	
**Level of education**				
MD, DMD, or Pharmacy students	161	53.5	9.17 (4.29)	0.072
BSc Students	140	46.5	10.04 (4.29)	
**Economic status**				
Low	22	7.3	9.18 (3.54)	0.302
Average	157	52.2	10.01 (4.30)	
Good	109	36.2	9.15 (4.08)	
Very Good	13	4.3	8.61 (4.23)	
**Father’s education**				
Primary school	68	22.6	9.20 (4.09)	0.129
Secondary school	54	17.9	9.14 (4.75)	
Diploma	90	29.9	9.24 (4.10)	
Academic	89	29.6	10.46 (3.86)	
**Mother’s education**				
Primary school	114	37.9	9.76 (3.90)	0.389
Secondary school	64	21.3	9.20 (4.92)	
Diploma	79	26.2	9.17 (4.10)	
Academic	44	14.6	10.36 (3.77)	

Table 2 shows mean (SD), range of score and bivariate associations among the predictor variables, most of which were statistically significant at either 0.05 or 0.01 level. The mean score of safe road-crossing behaviors was 9.57 [95% CI: 9.10, 10.05] and ranged from 0 to 16. For example, safe road-crossing behavior was associated with the attitude (r=0.648), subjective norms (r=0.525), prototype (r=0.244), willingness (r=0.268), and intention (r=0.200). Additionally, Intention was significantly related to the attitude (r=0.159), subjective norms (r=0.231), prototype (r=0.245), and willingness (r=0.207). Furthermore, willingness was associated with the attitude (r=0.267), subjective norms (r=0.364), and prototype(r=0.168). In addition, prototype was significantly related to the attitude (r=0.197), and subjective norms (r=0.202). Finally, subjective norms were significantly related to attitude (r=0.473).

**Table 2:** Coloration between different components of PWM.

Determinants	Mean (SD)	Range	X1	X2	X3	X4	X5
X1. Attitude	16.74 (4.95)	5-25	1				
X2. Subjective norms	14.81 (4.41)	5-25	0.473**	1			
X3. Prototype	9.79 (2.53)	3-15	0.197**	0.202**	1		
X4. Willingness	7.81 (2.55)	3-15	0.267**	0.364**	0.168**	1	
X5. Intention	6.15 (1.85)	2-10	0.159**	0.231**	0.245**	0.207**	1
X7. Behavior	9.57 (2.53)	0-16	0.648**	0.525**	0.244**	0.268**	0.200**

** Correlation is significant at the 0.01 level (2-tailed).

A linear regression analysis was performed to explain the variation in intention and willingness to adopt safe road-crossing behaviors. As can be seen in Table 3, attitude, subjective norms and prototype variables were statistically significant for predicting safe road-crossing behaviors: they accounted for 15% of the variation in willingness and 9% of the variation in intention to safe road-crossing behaviors. Furthermore, the findings of the current study suggest that between the willingness and intention variables, willingness was related more than intention to the safe road-crossing behaviors among the Iranian college students. 

**Table 3:** Linear regression analysis predicting willingness and intention to safe road-crossing behaviors by attitude, subjective norms and prototype.

Determinants	B	SE B	Βeta	t	P-value
**Willingness Path**	****	****	****	****	****
Attitude	0.057	0.031	0.110	1.808	0.072
Subjective norms	0.170	0.035	0.294	4.809	<0.001
Prototype	0.088	0.055	0.087	1.589	0.113
R2=0.15, F=17.633, P<0.001					
**Intention Path**	****	****	****	****	****
Attitude	0.014	0.024	0.038	0.607	0.544
Subjective norms	0.072	0.027	0.172	2.717	0.007
Prototype	0.148	0.041	0.203	3.577	<0.001
R2=0.09, F=10.443, P<0.001					
**Willingness and intention related to safe road-crossing behaviors**	****	****	****	****	****
Willingness	0.388	0.092	0.237	4.204	<0.001
Intention	0.340	0.127	0.151	2.681	0.008
R2=0.09, F=15.435, P<0.001					

## Discussion

This study was aimed to examine socio-cognitive determinants of safe road-crossing decisions and possible associated causative factors using PWM among KUMS students in west of Iran. The results of the present study indicate that willingness was related more than intention to the safe road-crossing behaviors among the Iranian college students. 

Our findings showed that the attitude, subjective norms, and prototype accounted for 15% and 9% of the variation in willingness and intention of safe road-crossing behaviors, respectively. In line with our findings, Evans and Norman showed that attitude and subjective norms accounted for 25% of the variation in road-crossing behaviors.^27^ Likewise, Zhou et al. showed the major socio-cognitive determinants of safe road-crossing behaviors were attitude and subjective norm in China in 2009.^13^ Furthermore, Andrews and colleagues have studied the association between cognitive determinants and the smoking prevention behaviors among children in 2008, reported that prototype was significant predictor of smoking prevention behaviors.^28^ In addition, a positive correlation between the PWM constructs was found. Safe road-crossing behavior was positively associated with attitude, subjective norms, prototype, willingness, and intention.

Our study showed that sex has a positive association with safe road-crossing behaviors. Similarly, Dıaz^29^ and Zhou et al.^13^ reported that females are more likely to engage in safe crossing behaviors and to appreciate the dangers of unsafe crossing behaviors. This difference is not merely due to the prolonged time that males spend in outdoor activities compared to females, but also that they are more prone to take greater risks. In fact, one potential explanation for this difference is that males are more likely to adopt both risky behavior and law-breaking both as drivers and as pedestrians and consequently are more involved in road-crossing injuries compared to females.^30^ For example, a study by Nasar and Troyer in 2013 suggested that pedestrian injuries related to mobile phone use were higher for males than females.^31^ Furthermore, we did not find a considerable association between age and safe road-crossing behaviors. However, by contrast, in the studies conducted by Bart et al.^32^ a significant correlation between age and safe road-crossing behaviors was found. Lack of a remarkable relationship between age and safe road-crossing behaviors may be due to the fact that our participants were aged between 19 to 30 years. Our results demonstrated that the level of participants’ education was not significantly associated with safe road-crossing behaviors. This might be due to all our participants’ being approximately on same educational level (medical and health education sector).

The findings of this research also revealed that economic status and parents' education were not associated with safe road-crossing behaviors. Plus, to the best of our knowledge, level of education had a potential effect on behaviors; while we observed no significant association between parents’ education and safe road-crossing behaviors. No explanation was provided for this finding. On the other hand, we found no significant association between economic status and safe road-crossing behaviors. This might be due to our participants’ easy access to health programs and campaigns and public media such as TV and radio regardless of their economic status.

At the end, variations across studies may be explained by the difference in the geographical locations, the ratio of males to females, sample size, and culture in the sample as well as differences in the instrument used for data collection and demographic characteristics. 

***Limitations***

The present study has several limitations. First, we used self-reported data to evaluate the cognitive determinants of safe road-crossing behaviors. These types of data may have a lower accuracy compared to interventional, observational or administrative data. Second, the nature of the study design (cross-sectional) did not allow further assessment of apparent associations over time. Third, our study was done in the KUMS in the west of Iran and the results of this study are not generalizable to other places and populations. Fourth, some students were unwilling to participate in the present study. 

Fifth, our study did obviously not study interactions between pedestrians and car drivers at intersections. Sixth, we did not investigate several variables such as vehicle speed, perceptual (perceptual-motor) limitations, driving experience, previous ‘near misses’ etc, which maybe affect safe road-crossing behaviors. Finally, cognitive determinants of behavior are unlimited, as PWM; however, we limited our focus to five constructs of behavior and did not examine constructs such as knowledge, beliefs and other important cognitive determinants of behavior. Hence, to have a better understanding of preventive behaviors, future studies are required to examine the other constructs of behavior and also the impact of educational interventions on safe road-crossing behaviors. Likewise, the selected age group in the present study is a potential limitation, and thus, to have a better understanding of safe road-crossing behaviors, it is suggested that future studies use appropriate age groups.

## Conclusion

In conclusion, the attitude, subjective norms, and prototype were the strongest predictors of safe road-crossing behaviors in KUMS students in west part of Iran. Therefore, more attention on these constructs is recommended to design health interventions and behavioral change programs. Moreover, sex is a significant predictor for safe road-crossing behaviors. Males are therefore greater risk takers and may take unsafe decision while crossing the road. Hence, pedestrians in Iran with the high incidence of RTAs need to be trained regarding safe road crossing behaviors and developing their skills to take the right decision while crossing the road, especially in vulnerable groups like the elderly and male pedestrians.

**Acknowledgment**

The authors gratefully acknowledge the contribution of the participants to the study and thank them for their help. Also, we would like to thank the Research Council of KUMS for the financial support.
